# *Clinacanthus nutans*: a review on ethnomedicinal uses, chemical constituents and pharmacological properties

**DOI:** 10.1080/13880209.2017.1288749

**Published:** 2017-02-15

**Authors:** Ihsan N. Zulkipli, Rajan Rajabalaya, Adi Idris, Nurul Atiqah Sulaiman, Sheba R. David

**Affiliations:** Pengiran Anak Puteri Rashidah Sa’adatul Bolkiah Institute of Health Sciences, Universiti Brunei Darussalam, Jalan Tungku Link, Gadong, Brunei Darussalam

**Keywords:** Sabah Snake Grass, phytochemicals, antitumorigenic, anticancer, anti-inflammatory, clinical trials, antiviral, antioxidant, flavonoids

## Abstract

**Context:** Medicinal plants have attracted global attention for their hidden therapeutic potential. *Clinacanthus nutans* (Burm.f) Lindau (Acanthaceae) (CN) is endemic in Southeast Asia. CN contains phytochemicals common to medicinal plants, such as flavonoids. Traditionally, CN has been used for a broad range of human ailments including snake bites and cancer.

**Objectives:** This article compiles the ethnomedicinal uses of CN and its phytochemistry, and thus provides a phytochemical library of CN. It also discusses the known pharmacological and biological effects of CN to enable better investigation of CN.

**Methods:** This literature review was limited to articles and websites published in the English language. MEDLINE and Google Scholar databases were searched from December 2014 to September 2016 using the following keywords: "*Clinacanthus nutans*" and "Belalai gajah". The results were reviewed to identify relevant articles. Information from relevant selected studies was systematically analyzed from contemporary ethnopharmacological sources, evaluated against scientific literature, and extracted into tables.

**Results:** The literature search yielded 124 articles which were then further scrutinized revealing the promising biological activities of CN, including antimicrobial, antiproliferative, antitumorigenic and anti-inflammatory effects. Few articles discussed the mechanisms for these pharmacological activities. Furthermore, CN was beneficial in small-scale clinical trials for genital *Herpes* and aphthous stomatitis.

**Conclusion:** Despite the rich ethnomedicinal knowledge behind the traditional uses of CN, the current scientific evidence to support these claims remains scant. More research is still needed to validate these medicinal claims, beginning by increasing the understanding of the biological actions of this plant.

## Introduction

Natural product drug discovery is a vibrant research area traversing nearly all scientific fields. Natural products, such as medicinal plants, serve as a rich potential source of new therapeutic compounds (Ramesh et al. 2014). In many countries, traditional medicinal plants have been used to treat a plethora of ailments, and this precious ethnomedicinal knowledge has been passed on over many generations. For as long as they have inhabited the earth, humans have used plants as a medicinal source. Plant-based drug development has grown more sophisticated, with modern chemists using compounds isolated from plants as structural leads to generate novel compounds with additional benefits, such as lower toxicity or higher efficacy in drug-resistant diseases.

Most medicinal plants have been studied for a range of biological activities including anticarcinogenic, anti-inflammatory and antimicrobial activities. These activities were further evaluated to identify potential therapeutic benefits for various human ailments such as cancers, autoimmune diseases and chronic infections. Continuing the quest for plant-based natural products is critical because plants contain many potentially novel therapeutic compounds. *Clinacanthus nutans* (Burm.f) Lindau (Acanthaceae) (CN) has been selected for the focus of this review as the plant has garnered much attention from social media about its potential therapeutic benefits. CN is one of many medicinal plants traditionally used to treat various diseases and injuries such as skin rashes, burns, fever and snakebite (Aslam et al. 2015). Some cancer patients have claimed that CN leaf consumption has helped in treating their cancer and improving their health, although there is a lack of clinical studies to support these claims (Shim et al. 2013).

To undertake a systematic and extensive review, literature was searched from various computerized databases (MEDLINE and Google Scholar) up to September 2016 as available on PubMED. Moreover, this literature review was limited to websites and articles published in the English language. The following keywords were used: “*Clinacanthus nutans*”, “Sabah Snake Grass” and “Belalai gajah”. The results were reviewed to identify relevant articles. Contemporary sources of knowledge were also used to compare the ethnopharmacological information against the scientific literature available. Data from the selected studies was extracted systematically into tables for analysis. A total of 124 articles were found using the search method described above revealing promising biological activities of CN, including antimicrobial, antiproliferative, antitumorigenic and anti-inflammatory effects.

## *Clinacanthus nutans* botany

CN is known in different countries by various vernacular names listed in Table 1, such as *belalai gajah* in Brunei Darussalam and Malaysia. The plant is native to the tropical regions of Southeast Asia, particularly Thailand, and also grows in southern China and some temperate regions (Hanelt 2001). It is suggested in the Plant List A (2013) that the plant *Justicia nutans* Burm is synonymous with CN. *Clinacanthus siamensis* has also been identified as another name for CN; however, at least two studies and ‘The Plant List B’ (2013) has made a distinction between *C. siamensis* and CN (The Plant List B 2013; Kunsorn et al. 2013; Fong et al. 2014). Moreover, it is also confused with *Andrographis paniculata* (Burm. f.) Nees (Indian Snake grass), in the same family, because of their similar names (Fong et al. 2014).

**Table 1.** Vernacular names of *Clinacanthus nutans*.

Vernacular names	Language	Country	Reference
Belalai Gajah	*Malay*	*Malaysia, Brunei*	(P’ng et al. 2013)
Sabah Snake Grass	*English*	*Malaysia, Brunei*	(Arullappan et al. 2014)
Dandang Gendis, Gendis, Ki tajam	*Indonesian*	*Indonesia*	(Hariana 2013)
Sha Be She Cao, E zui hua, You Dun Cao	*Mandarin*	*China*	(Quattrocchi 2012) (Ying 2013)
Phaya Yo, Saled Pangpon Tua Mea	*Thai*	*Thailand*	(Watson & Preedy 2008; Quattrocchi 2012)

**Figure 1.**
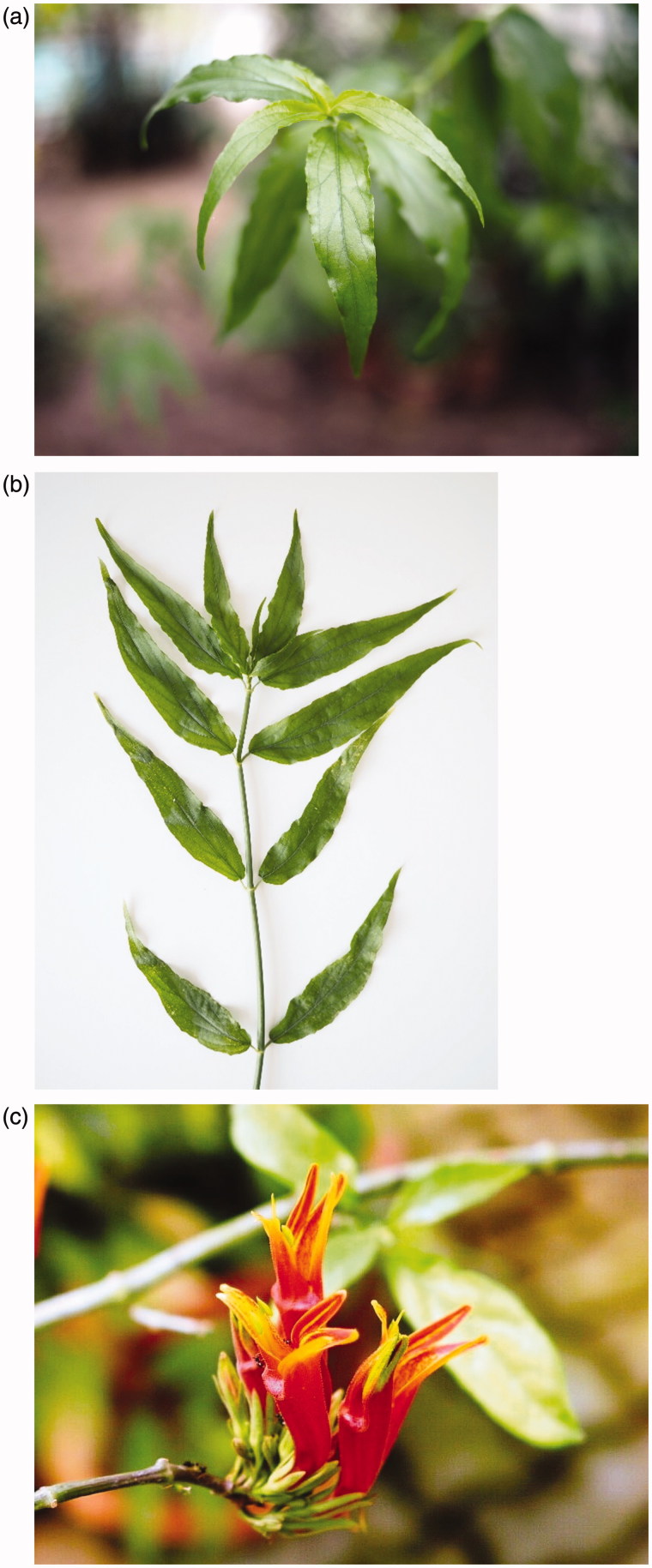
*Clinacanthus nutans* (a) leaves, (b) leaves and stem, and (c) flower.

The Acanthaceae family includes approximately 250 genera and 2500 species of dicotyledonous flowering plants that are mostly herbs and shrubs (Mohlenbrock 2008). CN is a shrub that can grow up to 1 to 3 m tall and has pubescent branches. Its stems are cylindrical, striated and smooth, while its leaves are lanceolate, long, narrow and oppositely arranged, and measure approximately 0.5–4 cm in width and 2.5–13 cm in length (Figure 1). The leaf has a pointed apex, and its margin is either exsculptate-dentate or subentire. The leaf base is rounded and pubescent on the nerves, and the length of the petiole is approximately 3–15 mm (Mat Ali et al. 2010).

CN has flowers that are arranged in a simple inflorescence (a cluster of flowers) called cyme at the tip of the branches, and each cyme has 5 to 8 flowers (Figure 1). The flower has a dark red corolla with a green base that measures approximately 4–6 cm. The lower lip of the flower has yellow streaks and is positioned upwards. The upper lip of the flower is located in the throat and is triangular with 2 stamens. The CN plant has a compacted 2-celled ovary with 2 ovules in each cell, and its style is filiform and shortly bidentate. Its capsule has an oblong shape and is basally contracted into a 4-seeded short, solid stalk and the seed is approximately 2 mm in diameter (Deng et al. 2002). The plant can be propagated through stem cutting (Mat et al. 2010).

Because CN leaves are widely sold as commercial products with the plant often harvested before it can flower, it has been suggested that the extensive propagation and harvest of the plant for commercial uses may hinder the plant from developing its sexual reproduction to maturity, thus resulting in a lack of flowers (Fong et al. 2014). Therefore, CN flowers are rare, and there is little information on how long it takes for the plant to flower and to fruit.

## Ethnomedicinal uses of CN

**Figure 2.**
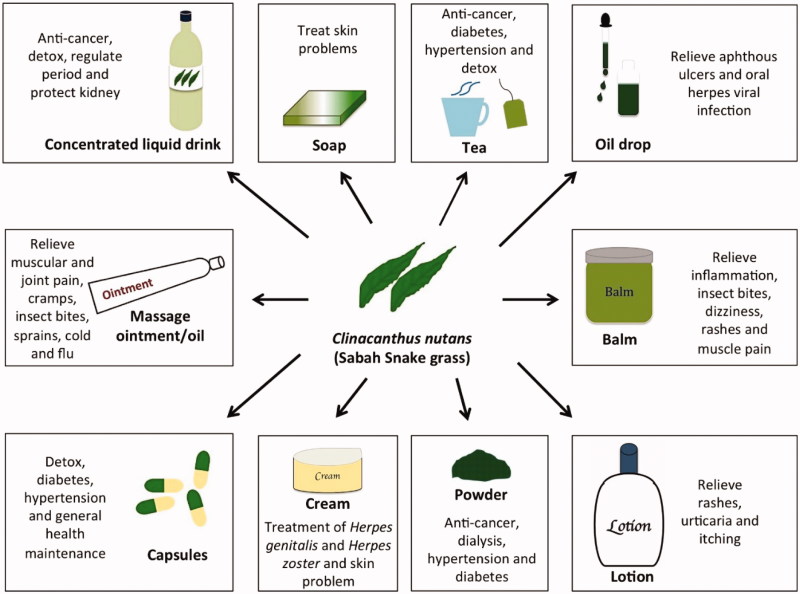
Commercial products made with *Clinacanthus nutans* available on the market.

CN is commonly used in traditional medicine in the Southeast Asian region, particularly in Thailand and Malaysia. Many traditional therapeutic uses for CN have been reported (Table 2), but clinical and scientific data support only a few of these. In general, only the leaves of CN are used in traditional medicine. The use of CN to treat *Herpes* virus infections is supported by the results of various scientific studies and clinical trials (Sangkitporn et al. 1993, 1995; Yoosook et al. 1999; Lipipun et al. 2011; Kongkaew & Chaiyakunapruk 2011; Kunsorn et al. 2013). CN leaves were initially consumed for general health in various countries in Southeast Asia (Shim et al. 2013; Siew et al. 2014). However, CN is gaining popularity in Malaysia and Singapore because of claims of its anticancer properties (Table 2). This has led to the availability of a wide variety of commercial products, including teas, drinks and powders (Figure 2).

**Table 2.** Traditional and modern uses of *Clinacanthus nutans*.

Indications	Plant part used	Herbal preparation	Prescription and dosage form	Reference
Anti-venom for snake, scorpion and insect bites	Fresh leaves	NAD	NAD	(Sakdarat et al. 2009; Sittiso et al. 2010; Kongkaew and Chaiyakunapruk 2011; Roeslan et al. 2012; Kunsorn et al. 2013; Rathnasamy et al. 2013; Arullappan et al. 2014)
Skin rashes	NAD	NAD	NAD	(Sakdarat et al. 2009; Sittiso et al. 2010; Kunsorn et al. 2013; Lau et al. 2014)
Varicella zoster, herpes simplex and herpes genitalis lesions	Fresh leaves	Ethanol extract	Topical use as a cream	(Direkbusarakom et al. 1998; Lipipun et al. 2003; Sakdarat et al. 2009; Sittiso et al. 2010; Roeslan et al. 2012; Kunsorn et al. 2013; Rathnasamy et al. 2013; Chelyn et al. 2014)
Pruritic rash	NAD	Ethanol extract	Topical use as a cream	(Chotchoungchatchai et al. 2012)
Aphthous ulcers	NAD	Ethanol extract in glycerin solution	Topical use as a cream	(Chotchoungchatchai et al. 2012)
Burns	NAD	Oil extract	Topical use as a cream	(Chotchoungchatchai et al. 2012)
Inflammation	Whole plant	NAD	NAD	(Rathnasamy et al. 2013; Arullappan et al. 2014; Ghasemzadeh et al. 2014)
Dysentery	Fresh leaves	Decoction of leaves boiled in water	Oral ingestion; Handful of fresh leaves is boiled in 5 glasses of water until water level reduces to 3 glasses. Dosage is 1 glass.	(Roosita et al. 2008; Roeslan et al. 2012; Arullappan et al. 2014; Globinmed 2015)
Diabetes	Fresh leaves	Decoction of leaves boiled in water	Oral ingestion; 7–12 fresh leaves boiled in 2 glasses of water, until water level reduces to 1 glass. Dosage is 1 glass, twice daily	(Roosita et al. 2008; Roeslan et al. 2012; Ching et al. 2013; Arullappan et al. 2014; Globinmed 2015)
Dysuria	Fresh leaves	Decoction of leaves boiled in water	Oral ingestion; 15g of fresh leaves boiled for 15 min. Dosage is once daily	(Roosita et al. 2008; Roeslan et al. 2012; Arullappan et al. 2014; Globinmed 2015)
Fever	NAD	NAD	NAD	(Lau et al. 2014)
Uric acid and gout	NAD	NAD	NAD	(Arullappan et al. 2014)
Urinates neuropathies and kidney syndrome	NAD	NAD	NAD	(Arullappan et al. 2014)
Cancer – liver, nasal cavity and general	Fresh leaves	Decoction of leaves boiled in water	Oral ingestion	(Roeslan et al. 2012; Arullappan et al. 2014; Aslam et al. 2015)
General health	Fresh leaves	Decoction of leaves boiled in water	Oral ingestion	(Lau et al. 2014; Aslam et al. 2015)
General health	Fresh leaves	Juiced with apple or sugarcane	Oral ingestion	(Shim et al. 2013)
General health	Dried leaves	Soaked in hot water	Oral ingestion	(Shim et al. 2013)
Skin rashes or scorpion and insect bites	NAD	NAD	NAD	(Ghasemzadeh et al. 2014)
Immunity boosting	NAD	NAD	NAD	(Siew et al. 2014)
General detoxification and health promotion	NAD	NAD	NAD	(Siew et al. 2014)
Prevention of cancer	NAD	NAD	NAD	(Siew et al. 2014)
Promoting bowel movements	NAD	NAD	NAD	(Siew et al. 2014)
Skin care	NAD	NAD	NAD	(Siew et al. 2014)
Benign growths	NAD	NAD	NAD	(Siew et al. 2014)
Cancer – general	NAD	NAD	NAD	(Siew et al. 2014)
Kidney problems	NAD	NAD	NAD	(Cancer cure 2015)
Cancer treatment	Fresh leaves	Juiced with apple and lemon	Oral ingestion; Number of leaves used for treatment of Cancer: Stage 1: 30 leaves per day Stage 2: 50 leaves per day Stage 3: 100 leaves per day Stage 4: 150–200 leaves per day	(Singapore Sabah Snake Grass/Clinacanthus Nutans/Belalai Gajah 2011)
High blood pressure and cholesterol	Fresh leaves	Juiced with apple and lemon	Oral ingestion	(Singapore Sabah Snake Grass/Clinacanthus Nutans/Belalai Gajah 2011)
Kidney problems and toxic urine	Fresh leaves	Juiced with red dates and black soybean	Oral ingestion	(Sabah snake grass 2009)
Urinary system syndrome, kidney problems and prostate inflammation	Fresh leaves	Juiced with green apple	Oral ingestion	(Sabah snake grass 2009)
Skin eczema, psoriasis and shingles	Fresh leaves	Decoction of leaves with stem and leaves of Rhinacanthus nasutus, then placed in bath water	Herbal bath	(Sabah snake grass 2009)
Skin eczema, psoriasis and shingles	Fresh leaves	Decoction of leaves with leaves of Polygonum Chinensis	Oral ingestion	(Sabah snake grass 2009)

NAD: Not appropriately described.

## Phytochemical review of CN

Dampawan et al. (1977) isolated lupeol and β-sitosterol crystals using ether and light petroleum solvent system, after having extracted the stem of CN in a Soxhlet apparatus with light petroleum. X-ray diffraction of these crystals revealed discrete molecules without short intermolecular contacts. Teshima et al. (1998) isolated sulfur-containing glucosides, clinacoside A and clinacoside B, from the colorless butanol soluble fraction of the methanol extract prepared from the CN stem and leaves. These phytoconstituents were identified using chemical and spectroscopic methods.

Based on reports that plant extracts had activity against the *Herpes simplex* virus (HSV), from pharmacological and clinical studies (Jayavasu et al. 2013), Janwitayanuchit et al. (2003) synthesized monoglycosyl diglycerides and investigated their structure–activity relationships. In this study, the fatty acyl and sugar moieties were identified as being critical for HSV inhibition and 1,2-*O*-dilinolenoyl-3-*O*-β-d-glucopyranosyl-*sn*-glycerol had the highest anti-HSV activity.

Sakdarat et al. (2006) extracted CN with chloroform and chromatographically separated different varieties of chlorophyll A and chlorophyll B (Sakdarat et al. 2006) and a total of eight compounds were isolated. Those related to chlorophyll A were 13^2^-hydroxy-(13^2^-*S*)-phaeophytin A, 13^2^-hydroxy-(13^2^-*R*)-phaeophytin A, purpurin-18-phytyl ester and phaeophorbide A. Those related to chlorophyll B were 13^2^-hydroxy-(13^2^-*S*)-chlorophyll B, 13^2^-hydroxy-(13^2^-*R*)-chlorophyll B, 13^2^-hydroxy-(13^2^-*S*)-phaeophytin B and 13^2^-hydroxy-(13^2^-*R*)-phaeophytin B. Additionally, trigalactosyl and digalactosyl diglyceride compounds were isolated from CN leaves and had anti-HSV activity.

Under similar conditions, the same group of researchers (Sakdarat et al. 2009) isolated three more chlorophyll derivatives of pheophytins, 13^2^-hydroxy-(13^2^-*R*)-pheophytin B, 13^2^-hydroxy-(13^2^-*S*)-pheophytin a and 13^2^-hydroxy-(13^2^-*R*)-pheophytin A. These compounds were characterized using ^1^H NMR and ^13^C NMR spectroscopy and had anti-HSV activity.

Using GC-MS, Yong et al. (2013) identified 14 chemical constituents (n-pentadecanol; eicosane; 1-nonadecene; heptadecane; dibutylphthalate; n-tetracosanol-1; heneicosane; behenic alcohol; 1-heptacosanol; 1,2-benzenedicarboxylic acid, mono(2-ethylhexyl) ester; nonadecyl heptafluorobutyrate; eicosayl trifluoroacetate; 1,2-benzenedicarboxylic acid, dinonyl ester; and phthalic acid dodecyl nonylester) from chloroform, methanol and aqueous leaf extracts of CN. The 1,2-benzenedicarboxylic acid mono (2-ethylhexyl) ester was the major chemical constituent, with a relative peak area of approximately 28.6% while those of the others were each less than 2%. Chelyn et al. (2014) identified C-glycosidic flavones, including isovitexin, vitexin, isoorientin, orientin and shaftoside, in an ethanol extract of CN leaves. These flavones were optimized and validated with an HPLC method for quantification and quality control of herbal materials.

Tu et al. (2014) isolated phytochemicals containing sulfur, clinamides A, B and C and 2-*cis*-entadamide A, from ethanol extracts of the aerial parts of CN. Clinamide A, a pale yellow oil, was assigned the molecular formula, C_6_H_11_NO_4_S by ^1^H-NMR spectroscopy and exhibited a methylsulfonyl signal and two methylene signals, including a trans-disubstituted double bond. Clinamide B was assigned a molecular formula of C_8_H_13_NO_4_S by HRESIMS (high-resolution electrospray ionization mass spectrometry), and its ^13^C-NMR spectrum was similar to that of entadamide C, but with an additional acetyl group. The partial NMR spectrum of clinamide C, a pale yellow oil, also indicated a close structural similarity to entadamide A and HRESIMS determined its molecular formula C_12_H_22_N_2_O_4_S_2_. HRESIMS assigned the molecular formula C_6_H_11_NO_2_S to both 2-cis-entadamide A and entadamide A, indicating that they are geometric isomers.

Yang et al. (2013) isolated saponins, phenolics, flavonoids, diterpenes and phytosterols from methanol extracts of CN leaves with a 15% w/w yield and with approximately 1.77 mg gallic acid equivalents per g of total phenolic content.

Use of CN leaves for various pharmacological purposes has increased exponentially because of information on the internet. Plant harvesting and preparation of its parts prior to extraction are of paramount importance, influencing the quantity and quality of the phytoconstituents extracted. Raya et al. (2015) assessed effects of storage duration on the phytochemical content of CN stems and leaves at different stages of harvesting. Phenolic content was 26% and 90% higher in younger leaves and stems, respectively, compared with their mature counterparts. Moreover, parts of mature plants had lower contents of phytochemicals, chlorophyll, and ascorbic acid compared with those from young plants. Also, prolonged storage reduced levels of these CN constituents. After storage for 4 d, the contents of total phenolics and chlorophyll were reduced to 50% and 25%, respectively, of the amounts in freshly prepared CN parts. Such evidence demands that fresh plant parts be used to avoid phytochemical loss and, thereby, optimize efficacy.

Huang et al. (2015) extracted dried aerial parts of CN using ethanol, purified the crude extract and identified compounds by HPLC with tandem mass spectrometry (LC/MS/MS). ^1^H-NMR analysis provided additional confirmation of compound identity. Flavonoids such as shaftoside, apigenin 6,8-C-α-l-pyranarabinoside, orientin, isoorientin, vitexin and isovitexin were observed in this study.

Several parameters such as solvent characteristics, prior plant preparation and thermal degradation influence the phytochemicals extracted from the plant. The extraction technique is a key factor determining the amounts and the natures of the phytoconstituents obtained. Mustapa et al. (2015) compared extraction efficiencies with microwave-assisted extraction (MAE), pressurized microwave-assisted extraction (P-MAE), supercritical carbon dioxide extraction (SFE) and the Soxhlet method. They reported on yields, extraction times and recovery of phytoconstituents, specifically, phenols, flavonoids, phytosterols and β-sitosterol. While MAE resulted in the highest yields of polyphenol and flavonoids, SFE was the best method for extracting phytosterols and β-sitosterol. P-MAE resulted in slightly improved yields of polyphenol and flavonoids. Overall, the study concluded that MAE was the most efficient extraction technique for CN, giving high extraction efficiency and better selectivity, compared with the other techniques, for compounds of nutraceutical interest, including those with anti-inflammatory, antioxidant and antimicrobial activities (Mustapa, Martin, Mato et al. 2015). The study further explored the influence of ethanol concentration and applied microwave energy and solvent-to-feed ratio on CN extraction using a microwave-assisted technique. Microwave pretreatment improved extraction rates by a factor of 2-5 fold, with water: ethanol (1:1) solvent (Mustapa, Martin, Gallego et al. 2015). Table 3 shows the chemical structures discussed in the phytochemistry section.

**Table 3.** Chemical structures of compounds isolated from *Clinacanthus nutans*.

Structure	Name	Reference
	13^2^-hydroxy-(13^2^-*S*)-phaeophytin a	(Sakdarat et al. 2009)
	(3β)-Lup-20(29)-en-3-ol	(Dampawan et al. 1977)
	beta-Sitoserol	(Dampawan et al. 1977)
	Clinacoside A	(Teshima et al. 1998)
	Clinacoside B	(Teshima et al. 1998)
	Shaftoside	(Chelyn et al. 2014); (Huang et al. 2015)
	Isoorientin	(Chelyn et al. 2014); (Huang et al. 2015)
	Orientin	(Chelyn et al. 2014); (Huang et al. 2015)
	Isovitexin	(Chelyn et al. 2014); (Huang et al. 2015)
	Vitexin	(Chelyn et al. 2014); (Huang et al. 2015)
	Isomollupentin 7-*O*-β-glucopyranoside	(Chelyn et al. 2014)
	Clinamide A	(Tu et al. 2014)
	Clinamide B	(Tu et al. 2014)
	Clinamide C	(Tu et al. 2014)
	2-*cis*-Entadamide A and Entadamide A	(Tu et al. 2014)
	Shaftoside (Apigenin 6-C-β-D-glucopyranosyl-8-C-α-L-arabinopyranoside)	(Huang et al. 2015)
	Apigenin 6,8-C-α-L-pyranarabinoside	(Huang et al. 2015)

Metabolomics generate metabolic fingerprints of an organism by identifying and quantifying its metabolites, which enhances the understanding of chemical variability among various organisms. Khoo et al. (2015) used nuclear magnetic resonance (NMR) to analyze the metabolite profile of CN leaves and stems, which were stratified based on two techniques: firstly, drying including air, oven, and freeze; secondly, extraction including soaking and sonication methods. Compared to leaves, stems contained a higher amount of terpenoids and phenolic compounds, correspondingly the activity levels of total phenolic content, α-glucosidase inhibition and antioxidant were higher as well, confirming to the well-established compound-activity correlation. Drying and extraction methods affect the yield of various phytoconstituents in extracts and thus its pharmacological activity. Partial least-squares analysis (PLS) biplot model analysis of the NMR revealed the superiority of oven and air drying methods over freeze drying and soaking methods for their yield of terpenoids, phenolic compounds, and glucosides, which were further confirmed by their corresponding better biological activities.

Huang et al. (2016) successfully attempted the purification and analysis of a novel polysaccharide-peptide complex from the leaves of CN, which showed significant promising results for gastric cancer cells SGC-7901 inhibition. The monosaccharide analysis, FTIR, 1H NMR and methylation analysis revealed the presence of CN polysaccharide which includes l-rhamnose and a backbone 1-6 linked Galp residues, while atomic force microscopy (AFM) displayed the entangled and branched structure of the compound. Identification of compounds will lead to the determination of the structure-activity relationship to develop it into a lead molecule. Multi-targeted therapy by plant extract as a single herb regimen either as the adjuvant or main treatment therapy calls for the systematic investigation of purified compounds of their beneficial therapeutic activities and mechanism to optimize them toward clinical studies.

## Pharmacological activities

CN is a medicinal plant with promising therapeutic potential. Many investigators have reported antioxidative (Pannangpetch et al. 2007; Arullappan et al. 2014), antiproliferative (Yong et al. 2013; Ghasemzadeh et al. 2014), antitumorigenic (Huang et al. 2015), antibacterial (Chomnawang et al. 2009; Arullappan et al. 2014), antiviral (Sangkitporn et al. 1993; Kunsorn et al. 2013) and anti-inflammatory (Sriwanthana et al. 1996; Wanikiat et al. 2008) effects of CN leaf extracts, as summarized in Table 4.

**Table 4.** *In vitro* and *in vivo* studies supporting pharmacological activities of *Clinacanthus nutans*.

Pharmacological assay	Extraction method	Observations	Bioactive compounds	References
***Antioxidant activity***				
2,2’-Azino-bis(3-ethylbenzothiazoline-6-sulphonic acid) (ABTS) Radical Cation-Scavenging Activity	Sonication with solvents of differing polarities†	• The 80% methanolic leaf extract gave the most potent ABTS radical scavenging activity, at over 60 mg GAE/g extract	• Protocatechuic acid • Chlorogenic acid • Ferulic acid • Caffeic acid	(Sarega et al. 2016b)
α-Glucosidase inhibition activity	Dried (freeze drying, oven drying or air drying) then extracted with ethanol (ultrasound assisted extraction or maceration)	• Leaf extracts had higher activity compared with stem extracts • Highest activity obtained with air-dried CN leaves extracted using ultrasound assisted extraction, at 37.34 ± 0.49% inhibition at 5000 ppm	• Gendarucin A • Gendarucin A isomer • 3,3-di-*O*-Methylellagic • cid • Schaftoside • Arabinosyl-glucosyl apigenin isomer • Ascorbin acid • Two isomeric oxoprolinates	(Khoo et al. 2015)
DPPH scavenging assay	Maceration in ethanol	• Maximum effect of 68% scavenging activity (approximately 0.08 times that of ascorbic acid control) • IC_50_ of 110 μg/ml	NR	(Pannangpetch et al. 2007)
	Methanol extraction, then dissolution in acetone*	• Low-scavenging activity compared with positive control	NR	(Wanikiat et al. 2008)
	Leaves were lyophilized, then sonicated in ethanol	• Significantly lower activity than corresponding green tea extracts	• Polyphenols	(Yuann et al. 2012)
	Chloroform, methanol and aqueous extraction, after grinding	• Highest antioxidant capacity with chloroform extraction (7853 μg Teq/g extract), where Teq is defined as the Trolox equivalent • Lowest antioxidant capacity with aqueous extraction (864 μg Teq/g extract)	• Phthalic acid mono (2-ethylhexyl) ester	(Yong et al. 2013)
	Petroleum ether, ethyl acetate and methanol extraction, after pulverization†	• Highest activity with petroleum ether extraction (82% scavenging activity at 4 mg/ml) • Second highest activity with methanol extraction (76% scavenging activity at 10 mg/ml) • Stem extracts showed weaker scavenging activities than leaf extracts	NR	(Arullappan et al. 2014)
	Hydrolysis, then dissolution in methanol‡	• Bud extracts showed higher scavenging activity than leaf extracts • Activity was higher in extracts from younger plants than from older plants; this may be related to phenolic content • Highest activity was shown in 1 y old buds with 66.2% DPPH scavenging and an IC_50_ of 64.6 μg/ml	NR	(Ghasemzadeh et al. 2014)
	Dried (freeze drying, oven drying or air drying) then extracted with ethanol (ultrasound assisted extraction or maceration)	• Leaf extracts had higher activity compared with stem extracts • Highest activity obtained with oven-dried CN leaves extracted using ultrasound assisted extraction, at 44.31 ± 3.16% scavenging activity at 5000 ppm	• Gendarucin A • Gendarucin A isomer • 3,3-di-*O*-Methylellagic acid • Schaftoside • Arabinosyl-glucosyl apigenin isomer • Ascorbin acid • Two isomeric oxoprolinates	(Khoo et al. 2015)
	Sonication with solvents of differing polarities†	• The 80% methanolic leaf extract gave the most potent DPPH radical scavenging activity, at 55.12%	• Protocatechuic acid • Chlorogenic acid • Ferulic acid • Caffeic acid	(Sarega et al. 2016b)
Ferric reducing antioxidant activity	Maceration in ethanol	• Activity is 59 times less potent than that of ascorbic acid	NR	(Pannangpetch et al. 2007)
	Hydrolysis, then dissolution in methanol‡	• Highest activity with 6 mo old buds (488 μM of Fe) • Significantly lower activity than that of the anti-oxidant standards, BHT and vitamin C	NR	(Ghasemzadeh et al. 2014)
	Sonication with solvents of differing polarities†	• The hot aqueous leaf extract gave the most potent ferric reducing activity, at almost 150 mg GAE/g extract	• Protocatechuic acid • Chlorogenic acid • Ferulic acid Caffeic acid	(Sarega et al. 2016b)
Galvinoxyl scavenging activity	Chloroform, methanol and aqueous extraction, after grinding	• Highest activity with chloroform extraction (12 249 μg Teq/g extract), where Teq is defined as the Trolox equivalent • Order of extract activities is: chloroform > methanol > aqueous	Phthalic acid mono (2-ethylhexyl) ester	(Yong et al. 2013)
Hydrogen peroxide scavenging activity	Chloroform, methanol and aqueous extraction, after grinding	• Relatively poor activity for all extracts • Highest activity was observed with methanol extraction, 35% scavenging activity at 100 μg/ml	Phthalic acid mono (2-ethylhexyl) ester	(Yong et al. 2013)
Inhibitory effect on PMA-induced free radical production by rat macrophages	Maceration with ethanol	• Free-radical production from PMA-stimulated macrophages was significantly reduced at extract concentrations of 30 μg/ml and higher	NR	(Pannangpetch et al. 2007)
Nitric oxide scavenging activity	Chloroform, methanol and aqueous extraction, after grinding	• Only the aqueous extract had nitric oxide scavenging activity; this was dose-dependent, with 32.3% scavenging activity at 100 μg/ml	Phthalic acid mono (2-ethylhexyl) ester	(Yong et al. 2013)
Protective effect on AAPH-induced hemolysis	Maceration with ethanol	• Extract showed a maximum inhibitory effect on hemolysis of 98%, with an IC_50_ of 359 μg/ml	• Phthalic acid mono (2-ethylhexyl) ester	(Yong et al. 2013)
Reducing power activity	Leaves were lyophilized, then sonicated with ethanol	• Showed significantly lower activity than corresponding green tea extracts	• Polyphenols	(Yuann et al. 2012)
Superoxide scavenging activity	Leaves were lyophilized, then sonicated with ethanol	• Showed almost 30-fold lower activity than corresponding green tea extracts	• Polyphenols	(Yuann et al. 2012)
Total phenolic content	Dried (freeze drying, oven drying or air drying) then extracted with ethanol (ultrasound assisted extraction or maceration)	• Leaf extracts had higher phenolic content compared with stem extracts • Highest activity obtained with air-dried CN leaves extracted using ultrasound assisted extraction, at 7.29 ± 0.11 mg GAE/g dw sample at 5000 ppm	• Gendarucin A • Gendarucin A isomer • 3,3-di-*O*-Methylellagic acid • Schaftoside • Arabinosyl-glucosyl apigenin isomer • Ascorbin acid • Two isomeric oxoprolinates	(Khoo et al. 2015)
***Anti-proliferative and cytotoxic activities***				
Allium cepa chromosome assay	Methanol and aqueous extraction	• Both extracts inhibited root growth in a dose-dependent manner, with the aqueous extract being more potent (EC_50_ of 630 mg/kg for the aqueous and 800 mg/kg for the methanol extracts) • Significantly higher incidence of chromosomal aberrations at 400 mg/kg with the aqueous extract Significantly higher incidence of cell death at 400 mg/kg with the methanol extract	NR	(Rathnasamy et al. 2013)
Cell lysis assay	Maceration in water	• Assay involved treatment of chick embryonic fibroblast cells with *Heterometrus laoticus* venom, which results in complete cell lysis within 20 min • Treatment for 30 min with 706 μg/ml extract resulted in approximately 50% cell viability • Treatment for 30 min with 406 μg/ml extract resulted in less than 10% cell viability recovery • However, treatment with 706 μg/ml or 406 μg/ml extract without pretreatment with venom resulted in 28% and 41% cell viability, respectively, indicating the cytotoxic effect of the extract	NR	(Uawonggul et al. 2006)
Hepatoma inhibition *in vivo*	Extraction with 30% ethanol¶	• Extract inhibited growth of HepA xenograft in mice • Treatment with extract for 10 d led to a significant and dose-dependent decrease in size and weight of tumour (8.2% and 58.6% decrease at doses 3 and 10 mg/kg, respectively) • Expression of proliferating cell nuclear antigen was markedly reduced in tumour cells • Expression levels of BAX and cleaved caspase-3 pro-apoptotic proteins were increased and levels of the anti-apoptotic Bcl-2 and p-AKT proteins were decreased, indicating apoptotic cell death	• Shaftoside • Isoorientin • Orientin • Isovitexin • Vitexin • Apigenin6-C-β-D-glucopyranosyl-8-C-α-L-arabiopyranoside 6,8-Apigenin-C-α-L-pyranarabinoside	(Huang et al. 2015)
MTT assay	Methanol extraction, then dissolution in acetone*	Human neutrophils • Concentrations up to 500 μg/ml did not affect neutrophil viability • Slight cytotoxic effect seen at 1000 μg/ml	NR	(Wanikiat et al. 2008)
	Methanol extraction	Saos-2 osteosarcoma cell line • Tested under normoxic and hypoxic conditions • Showed minimal toxicity under both conditions	NR	(Liew et al. 2012)
	Ethanol extraction via reflux, then sequential hexane and chloroform extraction using Soxhlet	Human gingival fibroblasts • Hexane extract did not affect proliferation • Chloroform extract was nontoxic only at concentrations lower than 400 μg/ml	NR	(Roeslan et al. 2012)
	Extracted consecutively with *n*-hexane and dichloromethane, then methanol	Vero cells • Highest activity shown with dichloromethane extract at 869 μg/ml	NR	(Kunsorn et al. 2013)
	Chloroform, methanol and aqueous extraction, after grinding	• Tested against various cancer cell lines • Aqueous extract significantly inhibited cell proliferation of HeLa and K-562 cells (36% and 41% inhibition, respectively) • Methanol extract showed no activity in NCI-23, HeLa, K-562 and Raji cell lines, weak activity to IMR32, SNU-1 and LS-174T cell lines and 42% inhibition in the HepG2 cell line at 100 μg/ml • Chloroform extract showed highest activity on K-562 and Raji cell lines at 100 μg/ml (91% with an IC_50_ of 48 μg/ml and 89% with an IC_50_ of 47 μg/ml, respectively)	Phthalic acid mono (2-ethylhexyl) ester	(Yong et al. 2013)
	Petroleum ether, ethyl acetate and methanol extraction, after pulverization†	HeLa • Petroleum ether leaf extract inhibited growth with an IC_50_ of 18 μg/ml K-562 • Petroleum ether leaf extract inhibited growth with an IC_50_ of 20 μg/ml	NR	(Arullappan et al. 2014)
	Preparation of crude methanol extract‡	HeLa • 6 month old bud extract significantly inhibited proliferation by 50% (IC_50_ of 56.8 μg/ml). • Extracts are nontoxic to a normal cell line at concentrations below 240 μg/ml	NR	(Ghasemzadeh et al. 2014)
	Maceration with polar solvents (methanol and dichloromethane) or non-polar solvents (hexane and diethyl ether) for three days at room temperature†	HEK-Blue™-hTLR4 and RAW264.7 • Viability of cells were not reduced after treatment with *CN* extracts compared to negative control • No morphological change observed in cells treated with *CN* extracts	Flavonoids	(Mai et al. 2016)
	Soxhlet extraction with chloroform	Vero cells • Purified compounds (see right) showed lower toxicity on Vero cells compared to crude chloroform extract • Highest CC_50_ was that of monogalactosyl diglyceride at 955 ± 7 μg/ml	• Monogalactosyl diglyceride • Digalactosyl diglyceride	(Pongmuangmul et al. 2016)
	Extraction in distilled water, precipitated with ethanol and fractionated using Superdex 200	RAW264.7 cells • Dose-dependent inhibition on cell proliferation on treatment with CN extract for 48 h	• Polysaccharide-peptide complex	(Huang et al. 2016)
***Anti-microbial activity***				
Disc diffusion assay	Ethanol extraction of tea leaves	• Performed both in the dark and with UV light exposure • Did not show significant antimicrobial activity against any microorganisms tested	NR	(Cheeptham & Towers 2002)
	Not described in paper§	• Extract did not show significant microbicidal activity against *Propionibacterium acnes* and *Staphylococcus epidermis*	NR	(Chomnawang et al. 2005)
	Maceration in ethanol	• No detectable activity against any microorganisms tested	NR	(Chomnawang et al. 2009)
Minimum inhibitory concentration (MIC) determination using microbroth dilution assay	Ethyl acetate extraction, after pulverization, then fractionation with gravity column chromatography	• Fraction with highest anti-microbial activity was F7, with MIC of 1.39 mg/ml against *Bacillus cereus*, *Escherichia coli*, *Salmonella enterica Typhium* and *Candida albicans*	NR	(Arullappan et al. 2014)
	Maceration in ethanol	• Showed no inhibitory activity against *Staphylococcus aureus* and *Staphylococcus epidermis*	NR	(Chomnawang et al. 2009)
	Maceration in methanol	• MIC was greater than or equal to 12.5 mg/ml for all organisms tested • *Staphylococcus aureus* and *Escherichia coli* showed the highest susceptibility with an MIC of 12.5 mg/ml	• Saponins • Phenolic compounds • Flavanoids • Diterpenes • Phytosterols	(Yang et al. 2013)
***Anti-viral activity***				
Anti-viral assay	Ethanol extraction, then preparation as a complex granule with polyvinylpyrolidone	• Extract showed anti-viral activity against yellow-head virus in black tiger shrimp, with an MIC of 1 μg/ml	NR	(Direkbusarakom et al. 1998)
Anti-dengue virus assay using Western blotting	Extraction in 80% ethanol, then fractionation using dichloroMethyl and methanol‖	• Moderate anti-dengue virus activity with an IC_50_ of 31.04 μg/ml	• Clinamide A • Clinamide B • Clinamide C 2-*cis*-Entadamide A	(Tu et al. 2014)
Inactivation assay	Preparation of crude methanol extract§	• Extract directly inactivated HSV-2 virus in a concentration dependent manner	NR	(Yoosook et al. 1999)
Patient meta-analysis	Extraction method is dependent on study	• Treatment with *C. nutans* topical cream results in improved "3-day-full-crusting" and "7-day-complete-healing" in patients with *Hepatitis genitalis* infections	NR	(Kongkaew and Chaiyakunapruk 2011)
Plaque inhibition assay	Not described in paper	• CN leaf extracts inhibited plaque formation by HSV-2 in baby hamster kidney cell line	NR	(Jayavasu et al. 1992)
	Aqueous extraction	• Extract did not show any activity against HSV-1 or HSV-2	NR	(Yoosook et al. 2000)
	Consecutive extraction with *n*-hexane, dichloromethane, then methanol	• All extracts showed over 50% inhibition of HSV-1 and HSV-2, activity at a 100 μg/ml concentration • Lowest IC_50_ values were obtained with the *n*-hexane extract against HSV-1, with IC_50_ of 32.05 ± 3.63 μg/mL and a selectivity index >50.36, and for the methanol extract against HSV-2, with IC_50_ of 65.13 μg/ml and a selectivity index >24.59	NR	(Kunsorn et al. 2013)
	Synthesis	• 1,2-O-dilinolenoyl-3-O-b-d-glucopyranosyl-sn-glycerol was identified as the most potent monoglycosyl diglyceride against HSV-1 and HSV-2 with IC_50_ values of 12.5 ± 0.5 and 18.5 ± 1.5 mg/ml, respectively	• Monoglycosyl diglycerides	(Janwitayanuchit et al. 2003)
	Sequential extraction with hexane, then chloroform; Four major fractions obtained by column chromatography	• Compounds 1, 2 and 3 showed 100% inhibition of HSV-1F virus activity (with IC_50_ values of 1.96, 3.11 and 3.11 nM, respectively) • Viral infection was inhibited at the step before viral entry	• Chlorophyll a Chlorophyll b • Related compounds	(Sakdarat et al. 2009)
	Soxhlet extraction with chloroform	• Post treatment of infected Vero cells showed 100% inhibition of plaque formation • Pretreatment with the compounds showed less than 50% plaque formation inhibition • Selectivity index (CC_50_/IC_50_) is highest for monoglactosyl duglyceride at 23.3 ± 0.9	• Monogalactosyl diglyceride • Digalactosyl diglyceride	(Pongmuangmul et al. 2016)
Protective efficacy in yellow-head disease in black tiger shrimp	Ethanol extraction, then preparation as a complex granule with polyvinylpyrolidone	• Test groups showed increased protection against yellow-head disease, compared with control group	NR	(Direkbusarakom et al. 1998)
Reverse transcriptase PCR	Sequential extraction with hexane, then chloroform	• Compound 2 (of 4) inhibited dengue virus 2 replication in A549 cells, with a 50% cytotoxic concentration (CC_50_) of 25 μg/ml • Treatment was effective pre-, but not post-incubation	• Chlorophyll a • Chlorophyll b • Related compounds	(Sittiso et al. 2010)
Yield reduction assay	Preparation of crude methanol extract§	• Virus titres were reduced to less than 2% of controls at the highest concentration tested	NR	(Yoosook et al. 1999)
***Anti-inflammatory activity***				
Carrageenan-induced hind paw oedema	Extraction in methanol and dissolution in acetone*	• Carrageenan-induced oedema was significantly reduced after 3 h by extract, in a dose-dependent manner	NR	(Wanikiat et al. 2008)
Elastase release	Extraction in 80% ethanol, then fractionation using dichloroMethyl and methanol‖	• Extract inhibited 68.33% of elastase release from human neutrophils at 10 μg/ml	• Clinamide A • Clinamide B • Clinamide C • 2-*cis*-Entadamide A	(Tu et al. 2014)
	Extraction in methanol and dissolution in acetone*	• Pre-incubation with extract caused weak but significant inhibition of elastase release, in a concentration dependent manner	NR	(Wanikiat et al. 2008)
Ethyl phenylpropiolate-induced rat ear oedema and myeloperoxidase (MPO) activity	Extraction in methanol and dissolution in acetone*	• Extract inhibited ear oedema formation in a dose-dependent manner • Most potent time point was 15 min • MPO activity was also significantly reduced at a dose of 9 mg/ear	NR	(Wanikiat et al. 2008)
Griess assay	Maceration with polar solvents (methanol and dichloromethane) or non-polar solvents (hexane and diethyl ether) for three days at room temperature†	• Lipopolysaccharide-stimulated nitric oxide generation measured in RAW 265.7 cells • All four *CN* extracts inhibited nitric oxide generation in a dose-dependent manner • Lowest IC_50NO_ (concentration at which extracts inhibited 50% of nitric oxide generation) was obtained with polar leaf extract (18.9 ± 3.6 μg/ml) • Also inhibits lipopolysaccharide-induced cytokine production – TNF-α, IFN-γ, IL-1β, IL-6, IL12p40 and IL-17	Flavonoids	(Mai et al. 2016)
TLR-4 activation assay	Maceration with polar solvents (methanol and dichloromethane) or non-polar solvents (hexane and diethyl ether) for three days at room temperature†	• Measures inflammation through the activation of toll-like receptor-4 • All four *CN* extracts inhibited TLR-4 activation in a dose-dependent manner • Lowest IC_50TLR-4_ (concentration at which extracts inhibited 50% of TLR-4 activation) was obtained with polar leaf extract (21.3 ± 5.0 μg/ml)	Flavonoids	(Mai et al. 2016)
***Modulation of immune response***				
Apoptosis of neutrophils	Extraction in methanol and dissolution in acetone*	• Extract had no significant effect on apoptosis rate in neutrophils	NR	(Wanikiat et al. 2008)
CD8+ T-cell infiltration	Extraction in 30% ethanol¶	• Extract caused enhanced CD8+ T-cell infiltration into hepatomas in HepA tumor-bearing mice, in a dose-dependent manner	• Shaftoside • Isoorientin • Orientin • Isovitexin • Vitexin • Apigenin6-C-β-D-glucopyranosyl-8-C-α-L-arabiopyranoside 6,8-Apigenin-C-α-L-pyranarabinoside	(Huang et al. 2015)
Cytokine production	Ethanol extraction	• IL-4 production increased at 2.5 mg/ml and 5 mg/ml, but the extract had no effect on IL-2 levels	NR	(Chompuki et al. 1996)
	Extraction in 30% ethanol¶	• Extract caused a dose-dependent increase in IFN-γ and IL-2 serum levels in HepA tumor-bearing mice • There were no changes in TNF-α and IL-10 serum levels	• Shaftoside • Isoorientin • Orientin • Isovitexin • Vitexin • Apigenin6-C-β-D-glucopyranosyl-8-C-α-L-arabinopyranoside • 6,8-Apigenin-C-α-L-pyranarabinoside	(Huang et al. 2015)
	Extraction in 80% ethanol, then fractionation using dichloroMethyl and methanol‖	• Concentrations up to 0.1 μg/ml led to upregulation of IFN-γ • Higher concentrations (>0.1 μg/ml) led to downregulation of IFN-γ	• Clinamide A • Clinamide B • Clinamide C • 2-*cis*-Entadamide A	(Tu et al. 2014)
Flow cytometry analysis	Ethanol extraction	• No effect of extract was observed on lymphocyte subpopulations	NR	(Chompuki et al. 1996)
Lymphocyte proliferation response assay	Ethanol extraction	• Lymphocyte proliferation was significantly increased at extract concentrations below 5 μg/ml and significantly decreased at those above 2.5 mg/ml	NR	(Chompuki et al. 1996)
Macrophage activation	Extraction in distilled water, precipitated with ethanol and fractionated using Superdex 200	• Macrophage activation was measured via production of nitric oxide • Incubation of RAW264.7 cells in CN extract for 48 hours showed a dose-dependent increase in the production of nitric oxide	Polysaccharide-peptide complex	(Huang et al. 2016)
Myeloperoxidase production	Extraction in methanol and dissolution in acetone*	• MPO production was significantly reduced by extract in a concentration dependent manner, with an IC_50_ of 219.5 μg/ml	NR	(Wanikiat et al. 2008)
Neutrophil chemokinesis assay	Extraction in methanol and dissolution in acetone*	• Neutrophil chemokinesis was significantly suppressed by extract in a concentration-dependent manner	NR	(Wanikiat et al. 2008)
Neutrophil chemotaxis assay	Extraction in methanol and dissolution in acetone*	• Neutrophil chemotaxis was significantly suppressed by extract in a concentration-dependent manner	NR	(Wanikiat et al. 2008)
NK cell activity assay	Ethanol extraction	• Significant reduction in NK activity at 1 mg/ml of crude extract and no detectable activity at 5 mg/ml of crude extract	NR	(Chompuki et al. 1996)
Superoxide anion generation	Extraction in methanol and dissolution in acetone*	• Superoxide anion generation was significantly reduced after incubation with extract for 10 min, in a concentration dependent manner, with an IC_50_ of 23.4 μg/ml	NR	(Wanikiat et al. 2008)
Th1 cell differentiation	Extraction in 30% ethanol¶	• Treatment of HepA tumour-bearing mice with extract increased proportion of IFN-γ+ CD4+ T cells (Th1) (15.4% vs. 4.6% in controls) • Treatment with extract also slightly decreased levels of IL-4+ CD4+ T cells (Th2) • IL-17A + CD4+ T cells and FOXP3+ CD4+ T cells remained unchanged	• Shaftoside • Isoorientin • Orientin • Isovitexin • Vitexin • Apigenin6-C-β-D-glucopyranosyl-8-C-α-L-arabiopyranoside 6,8-Apigenin-C-α-L-pyranarabinoside	(Huang et al. 2015)
***Acetylcholinesterase activity***				
*In vivo* assay in mice (Ellman method)	Maceration, then crude methanol extraction	• Acetylcholinesterase activity in Balb/C male mice liver, kidney and heart was significantly higher in the extract treated than in the control group • Acetylcholinesterase activity in the brain showed no significant differences between groups	NR	(Lau et al. 2014)
***Toxicity studies***				
*In vivo* subacute oral toxicity study in rats	Ethanol extract	• No signs of toxicity seen in mice after feeding the extract at the highest dose of 1.3g/kg of body weight • Platelet counts of rats fed with CN extract were significantly higher • However, creatinine levels were lower for CN-treated rats • No histopathological changes were detected	NR	(Chavalittumrong et al. 1995)
	Maceration with methanol	• No significant changes were seen in serum biochemical parameters, relative organ weight, body weight gain, food intake and water consumption with extract treatment • No noticeable signs of toxicity observed at any doses	NR	(P’ng et al. 2013)
	Maceration with water	• Sprague–Dawley rats were fed various doses of CN extract • No toxicity-related signs were observed in test animals after 28 days and 90 days • Treatment of test animals for 28 days with extract did not significantly alter haematological or biochemical parameters • Histopathological studies of mice organs did not show any pathological changes related to CN extract ingestion	NR	(Farsi et al. 2016)
*In vivo* assay in mice	Maceration, then crude methanol extraction	• No significant changes were seen in organ weight, food intake, water consumption and body weight in Balb/C male mice	NR	(Lau et al. 2014)
DNA integrity test	Lyophilized, then sonicated with ethanol	• Decreased cleavage of supercoiled plasmid DNA in *Escherichia coli* • Protective effect of CN extracts lasts longer than effect of green tea extracts	• Polyphenols	(Yuann et al. 2012)
Mutagenic effect (Ames test)	Maceration with water	• CN extract did not increase the number of mutant bacterial colonies in the *S. typhimirium* mutagenicity assay	• NR	(Farsi et al. 2016)
Hypoxia-induced toxicity assay	Ethanol extraction	• Treatment with CN extracts increased viability of primary neurons by 61% after exposure to hypoxia	• NR	(Tsai et al. 2016)
***Protection against insulin resistance and hyperlipidaemia***				
Measurement of insulin resistance biomarkers	Sonication with 80% aqueous methanol and water	• Mice treated with CN extracts showed slower weight gain caused by a high fat, high cholesterol diet • Significantly lower fasting blood glucose in mice treated with CN extracts, with increased insulin sensitivity • Decreased levels of biomarkers of obesity and insulin resistance in mice treated with CN extracts – RBP4, adiponectin, leptin, IL-6 and TNF-α	• Protocatechuic acid • Chlorogenic acid • Caffeic acid • Cinammic Acid (in methanol extract) • Ferullic acid (in methanol extract)	(Sarega et al. 2016a)
Measurement of weight gain and lipid profile in rats	Extracted with solvents of differing polarities: hexane, ethyl acetate, 80% methanol, water, and hot water (70^∘^C)†	• Extracts of CN slowed rate of weight gain in Sprague–Dawley rats fed a high-fat diet • Improvements in markers of hyperlipidaemia-associated stress after supplementation with CN extracts • Improvements in antioxidant enzyme activities in serum after supplementation with CN	• Chlorogenic acid • Cinnamic acid • Ferulic acid • Vanillic acid • Gallic acid • Caffeic acid	(Sarega et al. 2016b)
***Antinociceptive activity***				
Acetic acid-induced abdominal constriction test, hot plate test, formalin-induced paw licking test,	Maceration in methanol	• Treatment of mice with *CN* extracts showed that the extracts have an antinociceptive effect in all three of the antinociceptive assays carried out ^ Reduced abdominal writhing due to attenuation of imflammatory mediators ^ Delayed response to hot plate ^ Decrease in formalin-induced paw-licking time	• Gallic acid • 4-Hydroxybenzoic acid • Caffeic acid • Coumaric acid • Schaftoside • Ferulic acid • Vitexin • Orientin • Forsythoside H • Isoorientin • Forsythoside I • Isovitexin • Diosmetin glycoside • Luteolin • Apigenin	(Abdul Rahim et al. 2016)

All extractions were carried out on CN leaves, except where denoted as follows:

* Twigs and leaves;

† stem bark and leaves;

‡ buds and leaves;

¶ not mentioned;

§ whole plant;

‖ aerial parts.

NR: Not reported.

### Antioxidant activity

Antioxidants are substances that neutralize potentially damaging oxidizing agents or free radicals, which are thought to cause chronic health problems in diseases such as cancer, cardiac disease, and aging-related disorders. Pannangpetch et al. (2007) reported that CN extracts significantly reduced oxidative free-radical production by phorbol 12-myristate 13-acetate (PMA)-stimulated rat macrophages. Furthermore, the extract showed a substantial inhibitory effect (98%) on haemolysis in a 2,2′-azobis(2-amidinopropane) dihydrochloride (AAPH)-induced cell lysis model. AAPH causes lysis of red blood cells through oxidation of lipids and proteins in the blood cell membranes. Subsequent studies demonstrated *in vitro* antioxidant effects of CN based on various criteria, as summarized in Table 4. Collectively, data from these studies showed clearly that CN extracts could have antioxidant properties.

### Antiproliferative and cytotoxic activity

Antiproliferative compounds are used to inhibit the growth of cells and can be potentially used in the treatment of cancer. CN leaf extracts were reported to inhibit proliferation of a number of cell types including HeLa (Yong et al. 2013; Arullappan et al. 2014; Ghasemzadeh et al. 2014) cell lines. The effects of CN extracts on these cell lines are summarized in Table 4. All these studies used the MTT assay as a standard measure of cell proliferation. This is a colorimetric assay measuring cellular metabolic activity. Few studies have tested the antiproliferative effects of CN on primary cell types, although one study tested CN extracts on primary human gingival fibroblasts and detected no antiproliferative activity (Roeslan et al. 2012).

### Antitumorigenic activity

While antiproliferative compounds exert their anticancer effects through the inhibition of cell proliferation (Zulkipli et al. 2015), compounds with antitumorigenic activities may have a myriad of effects, which may prevent the development, maturation or spread of cancerous cells. Only one study has shown the potential antitumorigenic activity of CN. A CN ethanol extract, compared with a control treatment, significantly reduced tumour growth in a mouse HepA hepatoma model (Huang et al. 2015). The antitumorigenic effect in this model was significantly greater than that of fluorouracil, an established chemotherapeutic drug. Western blotting analysis showed that high levels of the pro-apoptotic mediator, Bax and apoptotic executioner protein, caspase 3 in tumours extracted from CN-treated tumour-bearing HepA mice. This suggests that CN could induce apoptosis in cancer cells as a mechanism to halt cell proliferation during tumour growth. However, more conclusive data obtained in multiple types of tumour models will be needed to prove that CN has antitumorigenic activity.

### Antimicrobial activity

With the rise of antibiotic-resistant strains of bacteria in the clinical environment, scientists have turned to natural compounds in medicinal plants to identify potential new antibacterial compounds. The antibacterial effects of CN extracts have been tested on microbial strains (Yang et al. 2013; Arullappan et al. 2014). Several studies have reported that CN extracts inhibited bacterial growth and survival, while other studies have reported no antibacterial activities of CN extracts against similar species of bacteria (Table 4). Overall, these mixed findings suggest that the antimicrobial effects of CN extracts may be selective for only certain microorganisms. However, the exact mode of action of CN on bacteria killing is yet to be defined.

### Antiviral activity

One of the most common ethnomedicinal use for CN is for treating *Herpes* infections (Sangkitporn et al. 1993, 1995). It is not surprising that there has been much interest in identifying a potential anti-*Herpes* agent from CN. The early work by Jayavasu et al. (2013) showed that CN leaf extracts inhibited plaque formation by HSV-2 in a baby hamster kidney cell line, suggesting that these extracts may contain antiviral components. The majority of work on the antiviral effects of CN has focused on infections with HSV, the causative agent for genital *Herpes* in cell lines and HSV-infected animals (Table 4).

CN extract-based topical formulations were shown to be effective against the development and progression of skin lesions in a mouse model of cutaneous HSV-1 infection (Lipipun et al. 2011). Recently, Pongmuangmul et al. (2016) showed that purified monogalactosyl diglyceride (MGDG) and digalactosyl diglyceride (DGDG) from CN leaves resulted in 100% inhibition of HSV viral plaque formation. Glycoglycerolipids, such as MGDG, have previously been shown to exert anti-HSV effects (Janwitayanuchit et al. 2003). Although the mechanism of anti-HSV activity of these glycoglycerolipids is still not clear, the known antiviral effects of monoglycerides against enveloped viruses such as HSV (Thormar et al. 1994) makes these glycoglycerolipids potential drug candidates against HSV. Interestingly, work by several groups showed that compounds from CN leaf extracts were able to inhibit dengue virus activity (Sittiso et al. 2010; Tu et al. 2014). CN extracts were also shown to be effective against a fish virus, the yellow-head virus, in CHSE-214-infected cells (Direkbusarakom et al. 1998). This suggests that CN-derived products could be effective not only against viruses other than HSV but also against viruses from various animal hosts.

### Anti-inflammatory activity and immune-modulatory effects

Extracts from CN leaves have been used to reduce symptoms of inflammation in insect bites, *Herpes* infection and allergic responses in traditional medicine. A few reports have also described the effects of CN extracts on the immune system.

CN extracts at low doses increased peripheral blood mononuclear cells (PBMC) proliferation, suggesting potential mitogenic properties (Sriwanthana et al. 1996). Interestingly, levels of interleukin-4 IL-4, an anti-inflammatory cytokine, were elevated only at higher CN doses, suggesting that inflammatory effects could be dampened with such doses. However, in a hepatocarcinoma (HepA) tumour model in mice, IL-4 induction by CD4 + T-helper 1 lymphocytes (Th1 cells) was not affected by treatment with a CN ethanol extract, compared with vehicle (Huang et al. 2015). Th1 cells primarily secrete IL-2 and interferon (IFN)-γ, which can suppress tumour growth by promoting CD8 + cytotoxic T lymphocyte (CTL) function (Dunn et al. 2006). Indeed, treatment with a CN ethanol extract-induced IL-2 and IFN-γ release and promoted CD8 + CTL infiltration into the tumour tissue in the HepA tumour-bearing mice (Huang et al. 2015). This suggests that CN could modulate the adaptive immune system by skewing the immune system toward a Th1-biased response, which would favour tumour suppression. In contrast, CN was also shown to act on the innate immune system. In two rat models of inflammation, ethyl phenylpropionate-induced ear oedema, and carrageenan-induced hind paw oedema, CN extracts significantly reduced oedema in the ears and paws, respectively (Wanikiat et al. 2008). The same study also reported attenuation of *N*-formyl-methionyl-leucyl-phenylalanine (fMLP)-mediated migration (chemotaxis and chemokinesis) and function (myeloperoxidase production and elastase release) in neutrophils treated with CN extract. These studies suggest that bioactive compounds found in CN leaves may have multiple effects on the immune system and any resulting inflammation, depending on the model system used. Additional studies are summarized in Table 4, and key studies have been highlighted here.

### Toxicity studies

The purpose of toxicity studies as per Barle et al. (2012) is, ‘to determine the effect of an action on a biological system which can be used later to extrapolate the doses and effects on humans’. This data is essential to identify the optimal therapeutic dose and the highest dose up to which the extract can be given, above which lethality would be expected. The toxicity studies can be acute, sub-acute and chronic, where the former is the most commonly used type to evaluate the dose to be used for testing the dose required for preliminary testing.

Sub-chronic toxicity study for 90 days of ethanol CN extract exhibited similar food consumption in control and test yet body weight was significantly lower in male rats with 1 g/kg bw. Although toxicity was not observed at the given dose, platelet and creatinine levels were altered in different ways, with the former being higher than control (Chavalittumrong et al. 1995).

Sub-acute toxicity study for 14 days of methanol CN extract with a maximum 0.9 g/kg dose did not display any toxic effect in rats. Compounds administered by an oral route of administration are, in general, metabolized by the liver and eliminated by the kidney, thus are investigated during oral toxicological experiments. While AST and ALT are markers for hepatocyte integrity, blood urea and creatinine are biochemical indicators for renal function. Consecutive administration of the intervention substance in rats is equivalent to less than 7 days human consumption (World Health Organization 2004). ‘Acceptable daily intake is the level that is harmless to humans based on the non-observable adverse effect level value obtained from animal study’, as mentioned by Food and Agricultural Materials Inspection Center was calculated to be 9 mg/kg in humans (P’ng et al. 2013; FAMIC 2014).

The mice were orally administered 1000 mg/kg of methanol CN crude extract for 14 days and were observed to have normal behaviour related to central nervous system despite having significantly elevated levels of acetylcholinesterase enzyme in liver, heart and kidney but not in the brain (Lau et al. 2014). It would be beneficial to elucidate the compounds responsible and the mechanism of the elevated enzyme levels in various organs.

Farsi investigated the correlation between dose and exposure period and included male and female rats. The longer treatment duration, induced decreased ALP at 2000 mg/kg oral dose of aqueous CN, yet within the physiological range and is clinically insignificant. There was an increase in body weight on day 42. However, the contrary was true on day 77 in rats administered with 500 mg/kg. Nevertheless, significant changes were not observed in the group administered with 2000 mg/kg. Mutagenicity, the tendency of a test compound to induce DNA changes, was measured by the bacteria reverse mutation test and mutagenicity was not observed with CN (Farsi et al. 2016).

Methanol, ethanol and aqueous extracts of CN extracts were evaluated for toxicity in rats or mice to enrich the understanding of CN safety profile (summarized in Table 4). The animal toxicity experiments provide only preliminary information and must be followed by a battery of tests in animals. Subsequently, chronic and toxicokinetic assessments must be carried out to confirm the safety profile of CN.

## Clinical trials

In addition to *in vitro* work, small-scale clinical trials performed using a topical formulation of a CN extract showed significant resolution of clinical symptoms of genital *Herpes* (Sangkitporn et al. 1993) and *Herpes zoster* (Sangkitporn et al. 1995; Charuwichitratana et al. 1996), as compared with patients treated with a placebo. A meta analysis of patients with hepatitis genitalis and *Herpes zoster* infections indicated that treatment with creams containing CN extracts resulted in faster healing of infection-induced lesions (Kongkaew & Chaiyakunapruk 2011).

The review mentions about its three limitations, namely: inability to perform a test of publication bias, the influence of small study effect and overestimate of clinical trials effect due to non-blinding. Due to these limitations, the results are to be interpreted with caution and summons for well-designed, robust, systematic, randomized controlled trials, which is either double or triple blinded. However, use of acyclovir with CN with its potential synergistic effect beckons for use as an adjuvant therapy in H. genitalis treatment. Patients with minor recurrent aphthous stomatitis, which is benign mouth ulcer, were recruited for the randomized, double-blind controlled trial to assess the efficacy of CN (Buajeeb & Kraivaphan 1994). Three arms were recording the duration of ulcers and pain, with CN, triamcinolone acetonide, and placebo, wherein the interventions were given in Orabase^®^, an oral pain reliever. CN was better than placebo in shortening the ulcer duration than placebo, albeit triamcinolone acetonide was the best. Despite this evidence, the precise molecular mechanism of how CN extracts kill HSV is not known and warrants further elucidation in this area.

## Future directions

CN has been demonstrated to have pharmacological effects on both host cells and microbes. The potential use of CN as an anti-HSV agent is promising. Future studies should investigate the antiviral activity of CN against other types of viruses, especially those endemic to regions where CN is abundant. Studies on the molecular mechanism of how CN extracts kill HSV are also needed.

Research to date has shown the antibacterial activity of CN only *in vitro*, in minimum inhibitory concentration assays, and the mechanism of this antibacterial activity is still unknown. Also, effects of CN *in vivo* in animal infection models have not yet been demonstrated. The lack of observed toxicities when various oral doses of CN leaf methanol extracts were given to Sprague–Dawley rats (P’ng et al. 2013; Farsi et al. 2016), and Balb/c mice (Lau et al. 2014) suggested that testing CN in more *in vivo* studies is feasible.

The anti-inflammatory effects of CN suggest that it has the potential to modulate the immune system. However, there is little current evidence addressing this and studies measuring cytokine production in immune cells have conflicting results. Therefore, more investigations would be required to establish the mechanisms by which CN dampens inflammatory activity in immune cells.

The mechanism for anti-proliferation by CN also remains to be determined. A link between antioxidant activity and anticancer effects has been suggested, particularly for phenolic compounds, such as flavonoids, from medicinal plants (Kumar & Pandey 2013; Roleira et al. 2015). Therefore, it is possible that phenolic compounds from CN could possess such biological activities. Although *in vivo* studies showed that CN has potential as an anticancer agent, more evidence from additional *in vivo* tumor models will be required conclusively to prove the biological relevance of the anticancer properties of CN.

Most of the studies investigating the antiproliferative effects of CN extracts on cells have focused on using the MTT colorimetric assay. Alternative lines of investigation for future studies could include investigating whether the antiproliferative effects of CN involve a cell division blockade or initiation of a cell death pathway (Zulkipli et al. 2015). Interestingly, in a recent study, high levels of the pro-apoptotic mediator, Bax, and apoptotic executioner protein, caspase 3, were detected by western blotting in tumor tissue from HepA tumor-bearing mice treated with a CN extract (Huang et al. 2015). Such data suggest that CN might induce apoptosis in cancer cells as a mechanism to halt tumor growth. The reported potent antiproliferative and antioxidant activities suggest that CN could be a good source of anticancer therapies.

There is a discrepancy in the procurement and processing of CN leaves used for various purposes. It is interesting to note that most CN users prefer fresh leaves while most researchers have experimented with dried plant parts. Thus, more experimentation by researchers on the effects of storage conditions on extract efficacy is needed. Many websites including Singapore Sabah Snake Grass (2011) have described a regimen of treatment for cancer and diabetes with fresh CN leaves (Roosita et al. 2008; Singapore Sabah Snake Grass 2011; Ching et al. 2013; Globinmed 2015). Standardization of the chemical constituents present in the leaves, their interaction with chemical constituents present in other plants and the efficacy of CN-derived preparations for the different stages of cancer have yet to be confirmed. Moreover, researchers also need to consider the selection of plant parts as well as the method of extraction and suitable solvents, to isolate the chemical constituents present in CN fresh leaves. Further research on the harvesting of CN parts and their proper storage will be needed to minimize loss of essential phytochemicals. These isolated compounds should be analyzed to compare their efficacy with the CN preparations used traditionally. Although it has been claimed that ingestion of CN prevents cancer, its prophylactic action has not been demonstrated. These measures would be beneficial to the community of CN users. If a potent activity is identified, the common process of drug discovery may then be applied such as what has been done for *Viscum album* mistletoe plant (Lim et al. 2016). Any potential drug arising from CN extracts would need to be investigated to determine the best formulation, dosage and delivery route. Once the mechanisms of the antitumor and other pharmacological properties of CN are better understood, it will be possible to identify molecular targets for upstream drug discovery research.
